# Evidence of Microglial Immune Response Following Coronavirus PHEV Infection of CNS

**DOI:** 10.3389/fimmu.2021.804625

**Published:** 2022-01-10

**Authors:** Jing Zhang, Zi Li, Huijun Lu, Junchao Shi, Rui Gao, Ying Ma, Yungang Lan, Jiyu Guan, Kui Zhao, Feng Gao, Wenqi He

**Affiliations:** ^1^ Key Laboratory of Zoonosis Research, Ministry of Education, College of Veterinary Medicine, Jilin University, Changchun, China; ^2^ Key Laboratory of Zoonosis Research, Ministry of Education, Institute of Zoonosis, Jilin University, Changchun, China

**Keywords:** microglia, porcine hemagglutinating encephalomyelitis virus, central nervous system, monocyte/macrophage, coronavirus

## Abstract

Porcine hemagglutinating encephalomyelitis virus (PHEV) is a highly neurotropic coronavirus that invades the host central nervous system (CNS) and causes neurological dysfunction. Microglia are key immune cells in the CNS, however, whether and how they response to PHEV infection remains unclear. Herein, microglial activation and proliferation were detected in the CNS of PHEV-infected mice, as along with the proinflammatory response. Moreover, the production of proinflammatory cytokines induced by moderately activated microglia limited viral replication in the early stage of infection. Microglial depletion assays showed that during late infection, excess activation of microglia aggravated neurological symptoms, BBB destruction, and peripheral monocyte/macrophage infiltration into the CNS. Using an *in vitro* brain slice model, PHEV was identified to specifically and moderately induce microglial activation in the absence of peripheral immune cells infiltration. Consistently, macrophage clearance from circulating blood indicated that peripheral monocytes/macrophages crossing the BBB of mice were responsible for excess activation of microglia and CNS damage in late PHEV infection. Overall, our findings provide evidence supporting a dual role for microglia in the host CNS in response to coronavirus PHEV invasion.

## Introduction

Porcine hemagglutinating encephalomyelitis was first reported in Ontario, Canada, in 1958, and porcine hemagglutinating encephalomyelitis virus (PHEV) was subsequently isolated from the brains of sucking piglets with neurological disorders (1). Serological tests indicated a global PHE pandemic whose typical clinical manifestations include severe encephalomyelitis, vomiting and wasting disease (VWD), and influenza-like illness (ILI) (2, 3). PHEV is a member of the neurotropic β-coronavirus family, together with severe acute respiratory syndrome coronavirus 2 (SARS-CoV-2) and mouse hepatitis virus (MHV) (4, 5). Similar to rabies virus (RABV) and pseudorabies virus (PRV) (6, 7), PHEV initiates a peripheral nervous system infection and spreads transsynaptically to the central nervous system (CNS), leading to inflammation or neurodegenerative disease (8–10).

Microglia, important innate immune cells within the CNS, play multifaceted roles in neural-immune interactions and confront pathogen invasion (11). Activation of microglia occurs on a continuum between pro-inflammatory (M1) and anti-inflammatory (M2) extremely activated states (12). Microglial activation induced by viral infection usually leads to the production of a large number of proinflammatory cytokines that limit or promote viral replication and even neuronal damage (13–16). Virus-infected or injured neurons release adenosine triphosphate (ATP) into the microenvironment of the CNS, promoting microglial recruitment and activation through purinergic P2X7 receptor signaling for selective elimination (17). Certain pattern recognition receptors (PRRs) that are expressed at high levels on the surface of cell membranes and endosomes of microglia are responsible for recognizing viral DNA/RNA to activate the type I interferon signaling pathway, thus contributing to antiviral innate immunity (18–21). Complement C3 and its cleavage products released by virus-infected neurons are also recognized by microglia to activate phagocytose antigens, which affects memory impairment (22).

Unsurprisingly, some RNA viruses have evolved strategies to escape or take advantage of host innate immune responses. RABV escapes innate host defenses by reducing the immune response of microglia to maintain the integrity of the blood–brain barrier (BBB), thereby preventing the infiltration of peripheral immune cells into the CNS (23–25). In individuals with human immunodeficiency virus 1 (HIV-1)-associated neurocognitive disorders, the virus destroys the BBB and invades microglia, resulting in increased production of proinflammatory cytokines, followed by severe inflammation and neuron loss (26). SARS-CoV-2 infects neurons and activates microglia, especially in circumventricular organs (CVOs) lacking the BBB (27). Massive production of cytokines released by activated microglia protect the nervous system from SARS-CoV-2 invasion but induce an indiscriminate immune attack on each cell type in the CNS, especially neurons (28). In addition, microglia can eliminate viral infection through autophagy, which has been reported in Zika virus infection (29).

PHEV infection causes severe CNS injury by targeting neurons (30). Microarray assays in mice suggested that the mRNA expression of some genes associated with interferon signaling and the inflammatory response is significantly upregulated after PHEV infection (31). However, the role of microglia in innate immunity against PHEV in the CNS remains unclear. Here, we identified a dual role for microglia in neuroprotection and damage during PHEV infection. In the early stage of infection, moderate microglial activation restricts PHEV replication and distribution, while excess microglial activation leads to BBB destruction and peripheral monocyte/macrophage infiltration during late infection, thereby causing the rapid death of mice. An understanding of the double-edged sword function of microglial activation during PHEV infection provides better insights into neuroimmunological mechanisms, helping to develop immunomodulatory strategies for neurotropic coronavirus diseases.

## Result

### PHEV Stimulates Microglia Activation and Proliferation in the CNS

**Figure 1:**
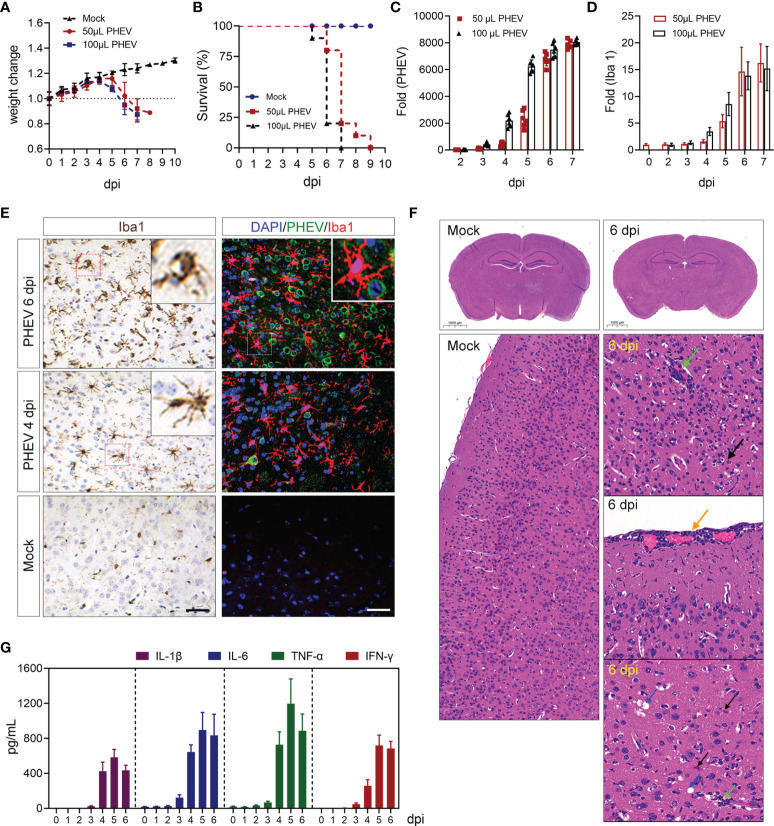
PHEV infection induces microglial activation and neuroinflammation. BALB/c mice were intranasally challenged with PHEV (10^4.15^/0.1 mL TCID_50_) at low dose and high dose, respectively. Weight changes **(A)** and survival **(B)** of PHEV-infected and control mice were recorded daily after infection. **(C)** Brain tissue was collected daily, and total RNA was extracted to evaluate viral RNA abundance (n=6). Fold change is relative to mRNA level at 2 dpi, when viral RNA could be detected in the cerebral cortex. **(D)** The expression of the Iba 1 mRNA was evaluated using qRT–PCR, with being relative to that at 0 dpi (n=6). **(E)** Brain biopsies from PHEV-infected mice collected at 4, 6 dpi were subjected to immunohistochemical staining for Iba 1 or immunofluorescence staining with Iba 1 (red) and PHEV-N (green) antibodies. Bars, 200 μm. **(F)** Histopathology of mouse brains was observed using H&E staining. Green arrow, inflammatory cell infiltration to form vascular cuffing. Yellow arrow, meningitis. Black arrow, injured neurons. Blue arrow, edema or cavity in the parenchyma and around blood vessels. **(G)** Secretion of cytokines was tested by ELISA (n=3).

Groups of 6-week-old female BALB/c mice were intranasally challenged with PHEV at a dose of 50 μL and 100 μL (10^4.15^/0.1 mL TCID_50_), respectively. PHEV-infected mice began to show ruffled fur, a hunched posture, and reduced activity; thereafter, significant weight loss was observed at day 5 postinfection (dpi) in high-dose group and 6 dpi in low-dose group (**Figure 1A**). All infected mice ultimately died, but the survival duration was shorter during high doses of infection (**Figure 1B**). PHEV- or mock-infected mice were sacrificed within 7 day covers the entire course of disease, and then brain tissues were collected for qRT–PCR analysis to assess viral replication and the dynamics of microglial activation. Substantial increases in viral RNA and microglial marker Iba 1 mRNA abundance were detected in the cerebral cortex when PHEV invasion (**Figures 1C, D**), although there are slight differences between brain regions (**Supplementary Figure 1**). Immunohistochemically, we found massive accumulation of activated microglia characterized by an amoeboid shape, protuberant retraction, and cell body hypertrophy, following CNS infection (**Figure 1E**). Immunostaining of brain sections indicated that PHEV was located mainly in neurons and its neighboring microglia quickly changed from a resting state to an activated state after infection (**Figure 1E**). Histopathological observation showed visible CNS injury that was characterized by large quantities of inflammatory cell infiltration to form vascular cuffing (green arrow) and meningitis (yellow arrow), injured neurons (black arrow), and edema or cavity in the parenchyma (blue arrow) (**Figure 1F**). In addition, large amounts of proinflammatory cytokines, including IL-1β, IL-6, TNF-α, and IFN-γ, were produced during PHEV infection, suggesting that inflammatory responses occurred (**Figure 1G**). Taken together, PHEV replicates in the brain and induces virus-specific microglial activation and proliferation in response to typical CNS damage.

### Microglial Activation Induces Inflammatory Responses in Early PHEV Infection

**Figure 2:**
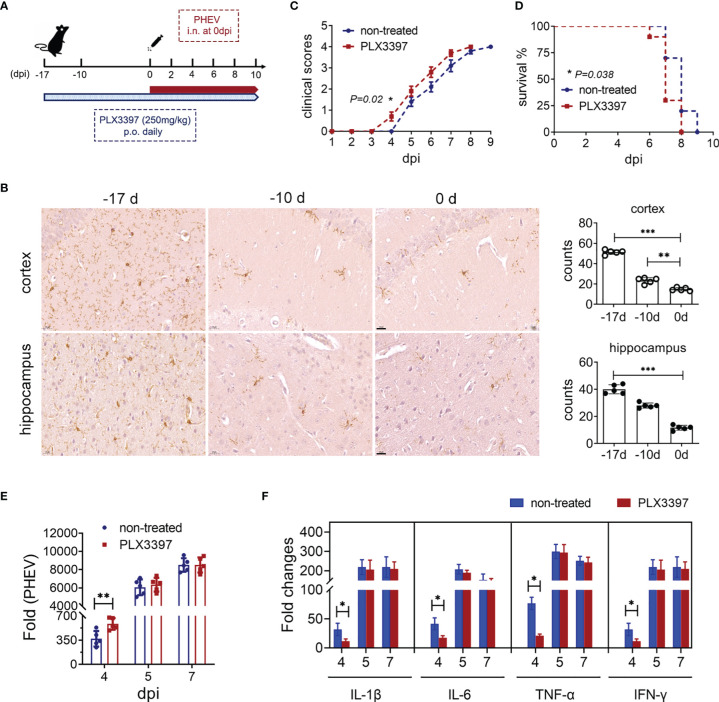
Administration of PLX3397 facilitates early infection of PHEV. **(A)** Procedure of PLX3397 administration and PHEV infection. **(B)** Microglial depletion was evaluated by Iba1 immunostaining. Iba1^+^ cells in the hippocampus and cortex were quantified in the right panels. Each column represents the mean ± SD for 5 fields in each group. Bars, 20 μm. **(C)** Clinical scores. **(D)** Survival curves. **(E)** PHEV mRNA expression was detected using qRT–PCR. **(F)** The expressions of IL-1β, IL-6, TNF-α, and IFN-γ were detected by qRT–PCR. Student’s t test (*, P<0.05; **, P<0.01; ***, P<0.001).

Mice were orally administered PLX3397, a CSF1R inhibitor, to deplete microglia and determine whether microglial activation is involved in PHEV infection. The procedure of PLX3397 treatment and PHEV challenge was performed as described in **Figure 2A**. IHC staining showed significant reductions in the number of Iba 1-positive microglia in both the cortex and hippocampus by more than 71% after 17 days of 250 mg/kg PLX3397 treatment (**Figure 2B**). Although, the clearance rate of microglia was higher in mice that treated with 290 mg/kg PLX3397 daily, but these mice suffer more weight loss and death (**Supplementary Figure 2**). Therefore, oral administration of 250 mg/kg PLX3397 was chosen to effectively remove microglia in the CNS. When intranasally challenged with PHEV, microglia-depleted mice developed clinical symptoms earlier than non-treated mice, but mortality was not affected (**Figures 2C, D**). At 4 dpi, the early stage of PHEV infection, microglial depletion enhanced viral replication but significantly reduced the mRNA expression of proinflammatory cytokines, including IL-1β, IL-6, TNF-α, and IFN-γ (**Figures 2E, F**). In contrast, there was no significant difference in proinflammatory cytokines at 5 and 7 dpi, that is, late infection. Therefore, we conclude that microglial activation induces the production of a large number of proinflammatory cytokines during the early stage of PHEV infection, which may contribute to limit viral replication.

### Excess Microglial Activation Is Accompanied by BBB Disruption and Macrophage Infiltration in Late Infection

**Figure 3:**
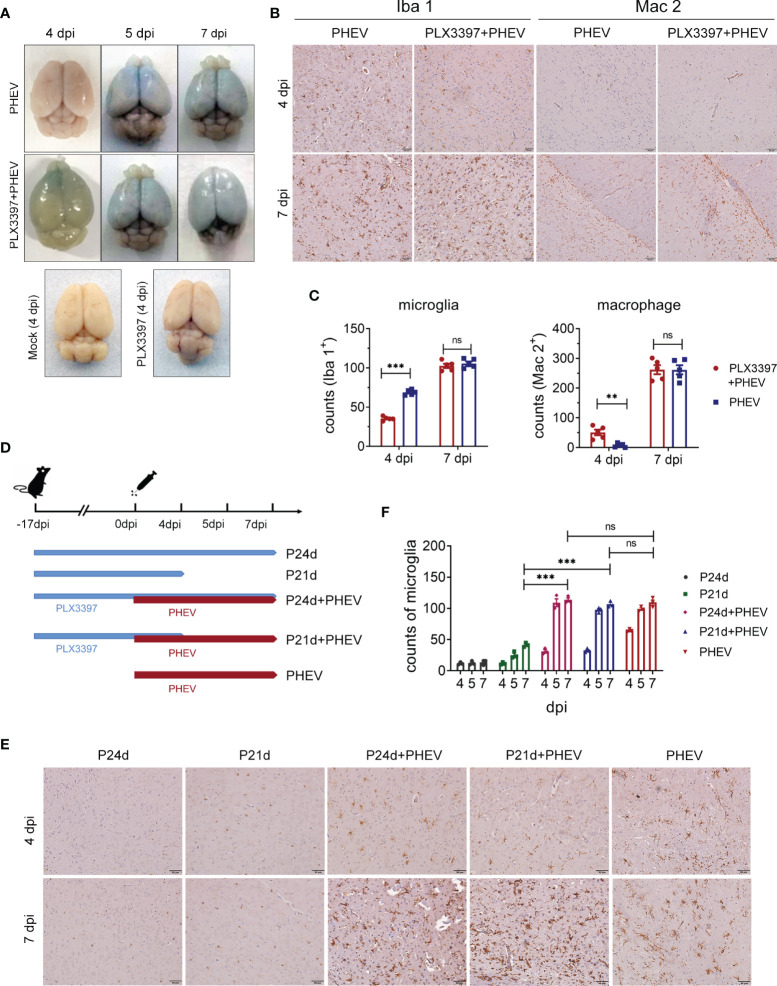
PHEV-specific excess microglial activation promotes BBB disruption and macrophage infiltration. **(A)** BBB destruction in PLX3397-administered mice was evaluated by i.v. injecting Evans blue (EB) at 4, 5, 7 dpi. **(B)** IHC staining of mice brain tissues. Microglia and infiltrated peripheral monocytes/macrophages were labeled with antibodies against Iba 1 and Mac 2, respectively. Bars, 2 μm. **(C)** Numbers of Iba 1^+^ cells and Mac 2^+^ cells. Five fields per group were analyzed. **(D)** Procedure of PLX3397 administration and PHEV infection. **(E)** IHC evaluation of microglial activation at 4 and 7 dpi. **(F)** Counts of Iba 1-labeled microglia. Statistical analyses were performed using Student’s t test (**, P<0.01; ***, P<0.001) ns, Not Significant.

Because PLX3397 failed to block the production of inflammatory cytokines at 5 to 7 dpi (**Figure 2F**), we tested the integrity of BBB during in late PHEV infection. At 4 dpi, increased BBB permeability was detected in PLX3397-treated mice using Evans blue tracing, which was one day earlier than in non-treated mice (**Figure 3A**). At this point, PLX3397 effectively inhibited microglial activation, although abundant Mac2-labeled monocytes/macrophages were detected (**Figures 3B, C**). At 7 dpi, both excessive activation of microglia and excessive infiltration of peripheral monocytes/macrophages synchronously occurred in the CNS (**Figures 3B, C**). Since PHEV-infected mice exhibited a substantial loss of appetite after 4 dpi, we artificially stopped PLX3397 administration at 4 dpi to exclude the effect of differences in PLX3397 intake (**Figure 3D**). As shown in **Figures 3E, F**, microglia were almost completely depleted when mice were treated with PLX3397 for 21 days (P21d) at 4 dpi but recovered to more than 45% at 7 dpi compared to mice treated with PLX3397 for 24 days (P24d). These mice challenged with PHEV (P21d+PHEV, P24d+PHEV) always showed substantial microglial proliferation at 5 to 7 dpi, regardless of whether PLX3397 administration was stopped passively (**Figures 3E, F**). Thus, during late infection, PHEV-induced excess microglial activation aggravates brain damage and results in BBB destruction and peripheral monocyte/macrophage infiltration into the CNS.

### PHEV Specifically Drives Moderate Microglial Activation in a Brain Slice Culture Model *In Vitro*

**Figure 4:**
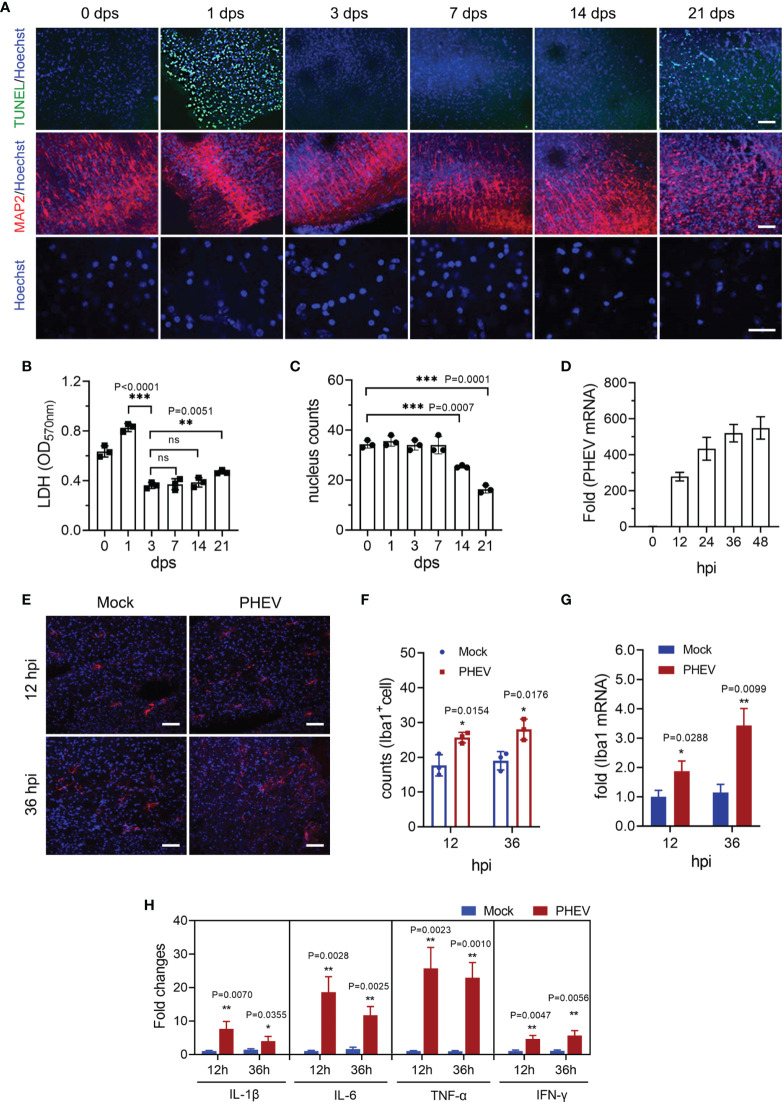
PHEV drives moderate microglial activation in a brain slice culture model. **(A)** Evaluation of the brain slice culture (BSC) model. Brain slices were collected at 1 to 21 dps. TUNEL, MAP2, and Hoechst staining were performed. Bars, 200 μm. **(B)** Counts of TUNEL-labeled nucleus in the BSC model. **(C)** LDH release in the supernatant was detected daily. **(D)** PHEV mRNA was evaluated using qRT–PCR. **(E)** PHEV inoculation was performed at 3 dps and slices were subjected to immunofluorescence staining 12 and 36 h later. Bars, 200 μm. **(F)** Counts of Iba 1-labeled cells. **(G)** Iba 1 mRNA expression was detected using qRT–PCR at 12 and 36 hpi. **(H)** The levels of inflammatory cytokines were evaluated using qRT–PCR at 12 and 36 hpi. Student’s t test (*, P <0.05; **, P<0.01; ***, P<0.001). ns, Not Significant.

A PHEV-infected mouse brain slice culture (BSC) was introduced to exclude the effect of peripheral immune cells on microglial activation in the CNS. Coronal slices with a thickness of 300 μm prepared from 7-day-old mouse brain tissues were cultured *in vitro* and evaluated within 3 weeks. Although apoptosis was detected using TUNEL staining at 1 day postslicing (1 dps), it disappeared at approximately 3 dps until 21 dps, consistent with the level of lactate dehydrogenase (LDH) release in the culture supernatant (**Figures 4A, B**). Microtubule associated protein 2 (MAP2) and Hoechst staining showed neuron loss and remarkable karyorrhexis occurred after 14 dps, suggesting that the brain slice model was stable from 3 to 14 dps (**Figure 4C**). When challenged with PHEV at 3 dps for, the counts of Iba 1-labeled microglia and Iba1 mRNA expression were significantly increased as the virus infection progresses (**Figures 4D**–**G**). The detection of proinflammatory cytokines showed abundant secretion of IL-1β, IL-6, TNF-α and IFN-γ (**Figure 4H**), although lower levels were detected than those in the *in vivo* model (**Figure 1G**). Therefore, our data confirm that PHEV replication induces specific and moderate microglial activation in the CNS in the absence of peripheral immune cell infiltration, implying that the excess activation of microglia is attributed to macrophage infiltration.

### Macrophage Depletion Reduces Excess Microglial Activation and PHEV-Induced CNS Damage in Mice

**Figure 5:**
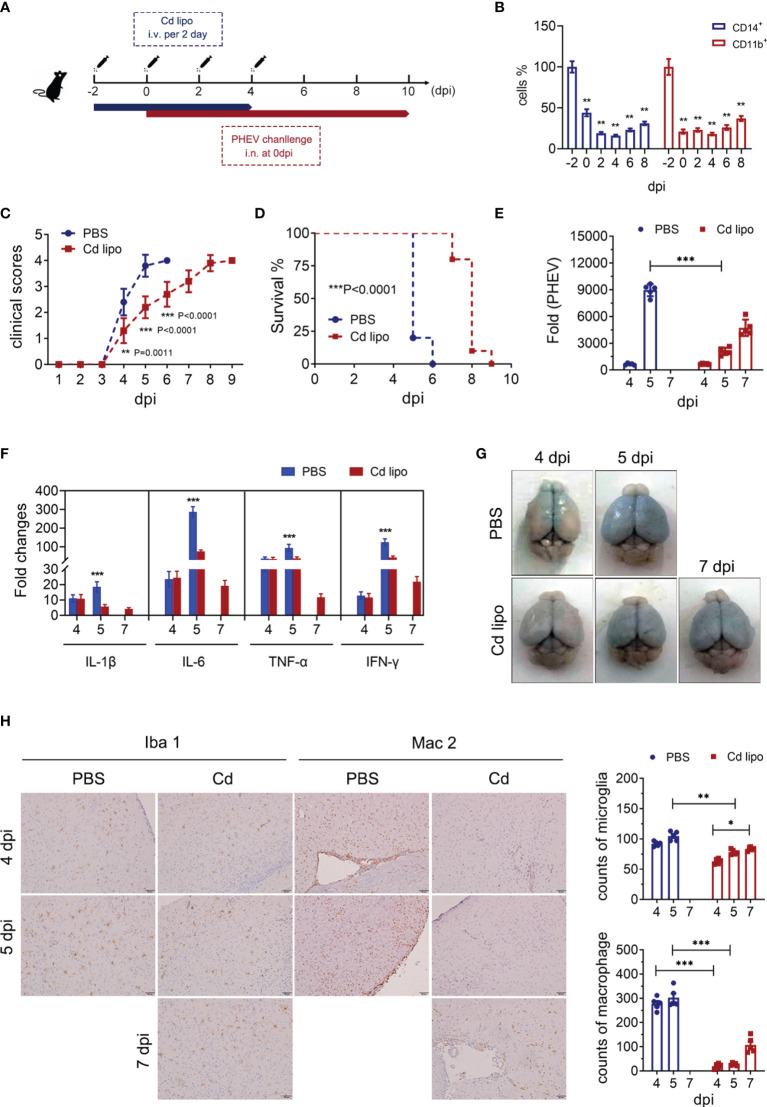
Macrophage depletion reduces microglial activation in late infection of PHEV. **(A)** Procedure of Cd lipos administration and PHEV infection. **(B)** The effect of Cd lipos on peripheral monocytes/macrophages was evaluated using flow cytometry. **(C)** Clinical scores. **(D)** Survival curves. **(E)** Viral replication in the brain was measured using qRT–PCR at 4, 5, and 7 dpi. **(F)** The expression of inflammatory cytokines, including IL-1β, IL-6, TNF-α, and IFN-γ, was evaluated at 4, 5, and 7 dpi using qRT–PCR. **(G)** BBB destruction in mice was evaluated by i.v. injecting EB at 4, 5, 7 dpi. **(H)** Morphological changes and microglial counts were analyzed at 4, 5, and 7 dpi. Microglia and peripheral monocytes/macrophages were labeled with antibodies against Iba 1 and Mac 2, respectively. Each column represents the mean ± SD of 5 fields per group (200×). Student’s t test (*, P <0.05; **, P<0.01; ***, P<0.001).

Clodronate liposome (Cd lipos) is a macrophage clearance tool used *in vivo*. To investigate the effect of peripheral immune cell infiltration on PHEV infection and microglial activation, mice are intravenously injected (i.v.) with Cd lipos to deplet peripheral monocytes/macrophages in circulation without significantly affecting microglial activation in CNS (**Figure 5A** and **Supplementary Figure 3**). Intravenous injection of Cd lipos and PHEV infection were performed as described in **Figure 5A**. Control mice were injected with PBS lipsomes, followed by PHEV challenge. Flow cytometry analysis revealed that approximately 80% of both CD14^+^ and CD11b^+^ monocytes/macrophages were consumed at 2 to 8 dpi (**Figure 5B**). Cd lipos-injected mice showed milder clinical symptoms and prolonged survival times, although mortality was not affected (**Figures 5C, D**). PHEV RNA replication was significantly inhibited by peripheral macrophage clearance (**Figure 5E**). Consistently, the expression of proinflammatory cytokines, including IL-1β, IL-6, TNF-α, and IFN-γ, was observably reduced at 5 dpi in Cd lipos-injected mice (**Figure 5F**). The Evans blue tracer analysis confirmed that Cd lipos did not alter the destruction of the BBB (**Figure 5G**). We next performed an IHC assay and found that Cd lipos treatment substantially inhibited Mac 2-positive monocyte/macrophage infiltration into the CNS and suppressed progressive microglial activation (**Figure 5H**). Based on these findings, circulating monocytes/macrophages that cross the BBB are responsible for excess microglial activation and CNS damage in late infection, and their depletion alleviates clinical symptoms, prolongs mouse survival, and restricts PHEV replication.

## Discussion

PHEV, MHV, SARS-CoV, and SARS-CoV-2 are notorious betacoronaviruses that have neuroinvasive capabilities. CNS infection with MHV induces an acute encephalomyelitis associated with focal areas of demyelination, while with the porcine coronavirus, PHEV, results in an encephalomyelitis associated with neurodegeneration. Pigs are the host of natural PHEV infection, but it is extremely difficult for us to use pigs in this study, for the doses of PLX3397 and Cd lipos are calculated depend on weights of experiment animal. PHEV artificially infected mice shows lethal severe encephalitis with less peripheral damage compared to both encephalitis and hepatitis caused by MHV invasion, suggesting that it is a promising neurotropic strain to model coronavirus-induced CNS pathogenesis. Moreover, the neurotropism of PHEV in mice and nerve cells has been verified by many previous studies (32–34). Unlike PHEV, MHV attacks all types of CNS cells and peaks at 5 dpi, but is eventually cleared by CD8+T cells (35). CNS infections caused by SARS-CoV and SARS-CoV-2 are similar. The chronology of SARS-CoV-2 infection can process neurological symptoms after 5 dpi, before the onset of respiratory symptoms. Cytokine storm syndrome is one of the factors that patients with COVID-19 can develop acute cerebrovascular disease (36).

Microglia have been proven to play a critical antiviral role in many viral infectious diseases, but in some contexts, they also accelerate disease progression (37). Investigations of microglial functional states will inform about the roles in health and disease and contribute to a more precise understanding of the multifaceted roles of these resident immune cells in the CNS. In this study, we focused on microglia in the CNS and explored their roles in PHEV infection by altering the status and number of microglia. At the early stage of infection, PHEV promotes moderate microglial activation without inducing proinflammatory cytokine production in the mouse CNS. PLX3397 is a CSF1R inhibitor that widely used to deplete microglia in the CNS without affecting monocytes/macrophages infiltration into the CNS (38, 39). Although different doses of PLX3397 exert vary influences on circulating monocytes and peripheral tissue macrophages, PLX3397 stimulation was proved to effectively clear microglia in the spinal cord injury model, without significantly reduce peripheral immune cells infiltration (39, 40). In present paper, we administered PLX3397 to test the involvement of microglial activation in PHEV infection. The findings suggested PLX3397-mediated microglial depletion accelerates viral replication and aggravates neurological symptoms, indicating that early responses of microglia are beneficial to restrict PHEV replication. The acceleration of BBB destruction and monocyte/macrophage infiltration indicates that moderate microglial activation is not the key factor inducing CNS damage, instead may be related to the replication of PHEV during early infection. However, increased production of proinflammatory cytokines and excess microglial activation were observed in late PHEV infection. Termination of PLX3397 administration confirmed that excess microglial activation is closely related to BBB destruction and peripheral immune cell infiltration.

Peripheral monocytes, as infiltrating inflammatory immune cells, are crucial for CNS immune responses and play important roles in CNS infection. HIV-1 infects monocytes to activate microglia and contributes to severe neuroinflammation, which induces neuronal damage (41). However, in West Nile virus (WNV) infection, infiltrated monocytes prolong survival (42). Clearance of monocytes/macrophages from the peripheral blood by Cd lipos administration prolonged the process of PHEV infection and alleviated neurological symptoms, suggesting that peripheral monocyte/macrophage infiltration accelerated ongoing immunopathological processes. Unfortunately, peripheral monocytes depletion did not completely protect the PHEV-infected mice from death. Unlike HIV-1 (43), PHEV does not infect microglia, but infiltrated macrophages aggravate BBB destruction and neural damage by promoting rapid and excessive activation of microglia and neuroinflammation. Alleviation of excess microglia activation driven by macrophage depletion displays a by-stander response to PHEV replication. Reduction of neuroinflammation and modest inhibition of viral replication are not key points to avoid the progress to severe CNS infection. Excessively activated microglia has been reported to be the main source of inflammatory cytokines and neurotoxic factors, which aggravate CNS diseases or even lead to death (13, 44, 45).

**Figure 6:**
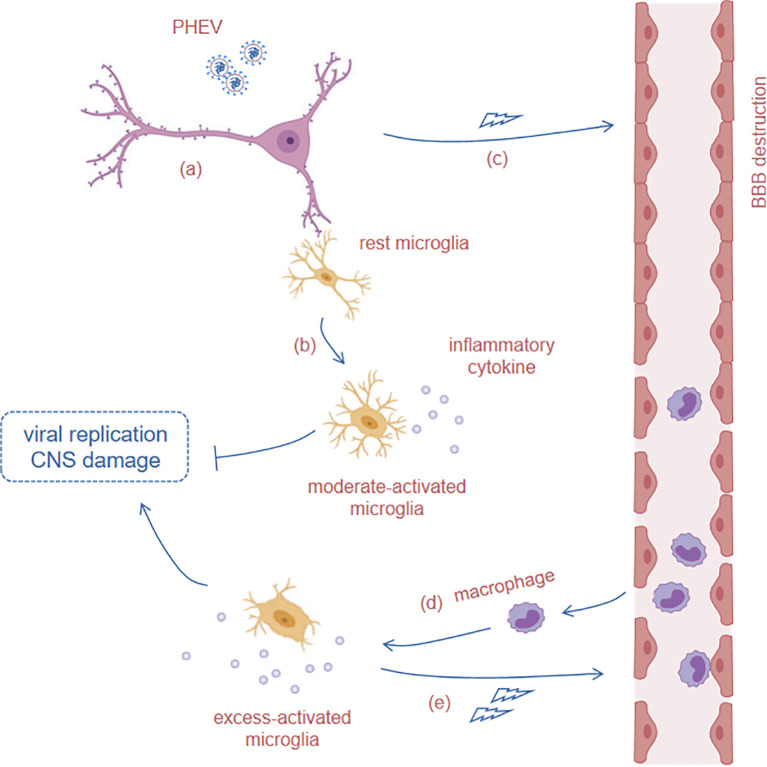
Microglial response acts in PHEV infection. PHEV infects neurons in the CNS, which release progeny virus and signaling molecules into the microenvironment. (a) PHEV infects neurons in the CNS. (b) In response to the signal of CNS infection, resting microglia are moderately activated and produce inflammatory cytokines to restrict viral replication at the early stage (1 to 4 dpi). (c) Meanwhile, rapid viral replication increases the permeability of BBB and recruits circulating monocyte/macrophage to cross the BBB. (d) BBB destruction portends the late stage of PHEV infection (5 to 7 dpi), and the infiltrated monocytes/macrophages contribute to the excess activation of microglia and production of large amounts of inflammatory cytokines. (e) Excess microglial activation and inflammatory cytokines secretion further aggravate BBB destruction, thereby promoting peripheral monocyte/macrophage infiltration and CNS infection.

M1 activated microglia-mediated neuroinflammation is identified as an amplifier of virus-induced neuropathology during MHV infection (46). Here, we propose that excessively activated microglia are a strong signal for a poor prognosis of PHEV infection and then identify that they are driven by infiltrated macrophages and accelerate the process of infection and death. The functions of microglia in PHEV infection are depicted in **Figure 6**: (a) PHEV infects neurons in the CNS, which release progeny virus and damage signals into the microenvironment. (b) In response to CNS infection, resting microglia is moderately activated and initiate immune response, including inflammatory cytokines production, to restrict viral replication at the early stage (1 to 4 dpi). (c) Meanwhile, rapid viral replication increases the permeability of the BBB and recruits peripheral macrophages to cross the BBB. (d) BBB destruction portends the late stage of PHEV infection (5 to 7 dpi), and infiltrated macrophages contribute to excess activation of microglia and the production of large amounts of inflammatory cytokines. (e) Excess microglial activation and inflammatory cytokines production further aggravate BBB destruction, thereby promoting macrophages infiltration. This vicious cycle aggravates the clinical and neurological symptoms and accelerates the death of infected mice.

In summary, we identified two different statuses of microglial activation, and exposed microglia functioned as a double-edged sword by playing a neuroprotective role in moderately activated status to restrict viral replication at the early stage and a detrimental role in an excessively activated status to accelerate death at the late stage of PHEV infection. This regulatory mechanism of CNS microglia provides a theoretical basis to better understand the pathogenesis of PHEV infection and is meaningful to develop immunomodulatory strategies for CNS infectious or neurological diseases.

## Materials and Methods

### Virus

PHEV/CC14 (GenBank accession number, MF083115) was isolated in Jilin Province, China, and stored in the Molecular Pathology Laboratory (47). The viral titer was measured by calculating the TCID_50_ (10^4.15^/0.1 mL), and the virus was stored at -80°C until use.

### Animal Protocols

Three- to six-week-old BALB/c mice (male) were purchased and housed in an exclusive animal room with natural light. In the intranasal infection model, mice were first anaesthetized with a 0.5% sodium pentobarbital solution (0.15 μL/10 g) to ensure the efficiency of viral challenge, and then 50 μL or 100 μL of PHEV stocks were slowly instilled into the nasal cavity with microsyringe needles.

### Clinical Score

Clinical symptoms were scored according to the process of infection and clinical symptom characteristics as follows: Score 0, normal activities and normal diets; Score 1, reduced activities and decreased appetite; Score 2, untidy hair with a curled up and arched back, loss of appetite, neurological symptoms including tics and squeaks, etc., and sound and touch sensitivity; Score 3, severe neurological symptoms with a high degree of tics and squeaks, dog sitting and leaning back with poor balance, paralysis, and extreme emaciation; Score 4, death. The clinical score was recorded once a day and is presented in a broken line graph.

### Quantitative Reverse Transcription PCR

The cerebral cortex of brain tissue was quickly harvested after the mice were anaesthetized. Total RNA was extracted using RNAiso reagent (Takara), and cDNAs were acquired using reverse transcription with PrimeScript reverse transcriptase (Takara). Expression of mRNA was assessed using quantitative reverse transcription PCR (qRT–PCR) with a SYBR qPCR kit (Biomake), and beta-actin served as a housekeeping gene for normalization. Reactions were performed under the following conditions: 95°C for 10 min, 40 cycles of 95°C for 15 s and 60°C for 30 s, with a final step of 72°C for 5 min. Relative difference was calculated as fold expression using the equation 2^-ΔΔCt^. Primer sequences that were used in this paper were designed as follows: PHEV-S, forward: 5’-GGG ACT TTC TAT GTT TTA-3’, reverse: 5’-ATA ATC AGC ATT CAC ATC-3’; IL-1β, forward: 5’-GTT CCC ATT AGA CAA CTG CAC TAC AG-3’, reverse: 5’-GTC GTT GCT TGG TTC TCC TTG TA-3’; IL-6, forward: 5’-CCA GAA ACC GCT ATG AAG TTC C-3’, reverse: 5’-GTT GGG AGT GGT ATC CTC TGT GA-3’; TNF-α, forward: 5’-GCA ACT GCT GCA CGA AAT C-3’, reverse: 5’-CTG CTT GTC CTC TGC CCA C-3’; IFN-γ, forward: 5’-TAG CCA AGA CTG TGA TTG CGG-3’, reverse: 5’-AGA CAT CTC CTC CCA TCA GCA G-3’; and beta-actin, forward: 5’-AGA GGG AAA TCG TGC GTG AC-3’, reverse: 5’-CAA TAG TGA CCT GGC CGT-3’.

### Brain Slice Culture

Organotypic brain slice cultures were generated using the method described in a previous study (48). Briefly, postnatal 7-day-old BALB/c mice were decapitated under sterile conditions, and whole brains were harvested without the cerebellum. Then, the brains were placed in Hank’s balanced salt solution (HBSS) and separated into two hemispheres. The brains were glued onto the chuck and sliced into 300 μm coronal sections using a vibratome (Leica VT1000A). The brain sections were carefully transferred to a 0.4 μm pore membrane (three slices per membrane) insert in 6-well culture plates, and 1 mL of culture medium was added to each well to ensure that each slice was cultivated at the interface between air and culture medium in a 37°C incubator with 5% CO_2_. The culture medium was composed of 50% MEM/HEPES (HyClone), 25% heat-inactivated horse serum (Ausbian), 25% HBSS (HyClone), 2 mM NaHCO_3_, 6.5 mg/mL glucose, and 2 mM glutamine. The brain sections were cultured for 3 weeks, and the medium was replaced every 2-3 d. PHEV chanllenge was performed at 3 dps. Brain slices were carefully rinsed by HBSS for 3 times and 20 μL of PHEV stocks were slowly added, followed by culturing in complete culture medium for indicated times.

### Hematoxylin and Eosin Staining

For histopathology, mice were anesthetized, and brain tissues were quickly removed and fixed with 10% neutral formalin for 24 h. The fixed brains were dehydrated in an orderly manner, cleared, and embedded in paraffin. Samples were cut into 5 μm coronal sections. Histopathology was evaluated by performing staining with hematoxylin and eosin.

### Immunohistochemistry

Brain sections were pretreated with heat-mediated antigen retrieval in sodium citrate buffer (pH 6.0) for 15-20 min, incubated with antibodies against Iba 1 or Mac 2 and incubated for 15 min at room temperature or overnight at 4°C. Immunochemical staining was performed using an IHC kit (MXb, CN) and DAB kit (MXb, CN) according to the manufacturer’s instructions. Mac 2-positive cells were analyzed using ImageJ software. Iba 1-positive microglial cells were counted artificially to exclude infiltrated macrophages according to the morphological characteristics.

### Immunofluorescence Staining

Paraffin-embedded brain tissues were sliced into 5 μm sections for indirect immunofluorescence staining. The sections were dewaxed, and heat-mediated antigen retrieval was performed with citrate buffer, pH 6.0. Then, the slices were blocked with 5% normal goat serum for 30 min and incubated with Iba1 and PHEV-N antibodies overnight at 4°C. After rinsing the sections with PBST, secondary antibodies were applied for 1 h at room temperature. Brain slice cultures were first fixed with 4% paraformaldehyde for 30 min and then treated with a MAP2 antibody diluted in PBS containing 1% Triton X-100 and 5% normal goat serum for 24 h at 4°C. After rinses with PBST containing 1% Triton X-100 3 times for 1 h, Alexa Fluor 594-conjugated anti-rabbit secondary antibodies were applied and incubated for 2 h at room temperature. Then, the slices were mounted with ProLong Gold Antifade Reagent containing Hoechst. The sections were observed, and images were captured using a confocal microscope (FV10-ASW 3.0, Olympus Europa Holding GmbH).

### PLX3397 Administration

Microglial depletion was conducted as described previously (49). Briefly, PLX3397 (pexidartinib; Meilunbio, China) was formulated in AIN-76A standard diets at a proportion of 250 mg/kg and was fed to three-week-old mice at a dose of 40 mg/kg/d consecutively. The efficiency of microglial depletion was determined by performing IHC with an Iba 1 antibody (Abcam, ab5076). Consecutive management for 17 days resulted in a greater than 80% depletion of microglia. Therefore, an infectious experiment was performed after 17 consecutive days of oral administration (p.o.) of PLX3397.

### Clodronate Liposome Administration

Clodronate liposomes (Cd lipos) and PBS liposomes (liposomal B.V.) were administered at a dose of 100 μL/10 g of body weight by i.v. injection through the tail vein every two days for four injections. The efficiency of peripheral monocyte/macrophage depletion by Cd lipos was determined by performing IHC with a Mac 2 antibody. After the second injection, the depletion efficiency was approximately 90%. Mice were challenged with PHEV at the time of the second injection.

### Evaluation of BBB Permeability Using Evans Blue Staining

Evans blue (EB) was prepared as a 1% solution diluted with 0.9% normal saline and administered by intravenous (i.v.) injection through the tail vein at a dose of 10 ml/kg of body weight (50). Mice were monitored for four hours to ensure that EB had fully circulated and was deposited systematically. Afterward, mice were anaesthetized and slowly perfused transcardially with 20 mL of ice-cold PBS, and the brain was separated and fixed with 10% neutral formalin for another four hours. Then, images were collected to observe the deposition of EB in brain tissue after crossing the BBB.

### Lactate Dehydrogenase Release Assay

In the brain slice culture experiment, cell necrosis or apoptosis was evaluated by measuring lactate dehydrogenase (LDH) release in the supernatant using an LDH Release Assay Kit (Beyotime, CN). The culture supernatant was harvested and quickly tested according to the instructions provided in the user guide.

### TUNEL Apoptosis Assay

The survival of cells in brain slice cultures was tested using a TUNEL Cell Apoptosis Detection Kit (WanleiBio, CN). Briefly, brain sections were fixed with 4% paraformaldehyde for 1 h and then blocked with 5% normal goat serum diluted in PBS containing 1% Triton X-100. The subsequent procedure was performed according to the instructions. The sections were observed, and images were captured using a confocal microscope (FV10-ASW 3.0, Olympus Europa Holding GmbH).

### Flow Cytometry

The efficiency of peripheral monocyte/macrophage depletion was evaluated using flow cytometry. Peripheral blood was collected and lysed with ACK lysis buffer to remove red blood cells. Cells were washed with PBS and stained with PE-conjugated CD14 or CD11b antibodies for 30 min at 4°C. Then, the cells were washed 3 times and resuspended in FACS washing buffer (PBS+1% BSA). Cells were examined using a FASCAria instrument (BD, USA) and analyzed using FlowJo 10.0 software.

### Statistical Analyses

All the data were evaluated using Student’s *t* test with GraphPad Prism, version 7.0 and are shown as the means ± SD (standard deviations). Significant differences between columns are indicated with asterisks, and a *P value* less than 0.05 was considered statistically significant.

## Data Availability Statement

The datasets presented in this study can be found in online repositories. The names of the repository/repositories and accession number(s) can be found in the article/**Supplementary Material**.

## Ethics Statement

The animal study was reviewed and approved by the Institutional Animal Care and Use Committee of the College of Veterinary Medicine, Jilin University.

## Author Contributions

JZ and WH designed and supervised the experiments. JZ, ZL, and HL performed most of the experiments and interpreted the data. JS recorded the clinical symptoms and mortality. RG and YM participated in the histopathology and immunohistochemistry analysis. JZ, HL, and YL supervised the project and interpreted the data. ZL, JG, and KZ wrote the main paper text. FG and WH revised the manuscript. All authors contributed to the article and approved the submitted version.

## Funding

This work was supported by the National Natural Science Foundation of China (grant 32172828 to WH, 31902262 to ZL, and 32172805 to FG), the Scientific and Technological Project of Jilin Province (grant 20210202041NC to WH), the Youth Science and Technology Talent Support Project of Jilin Province (grant QT202015 to ZL), and the Fundamental Research Funds for the Central Universities to ZL. The funders had no role in the study design, data collection and interpretation, or the decision to submit the work for publication.

## Conflict of Interest

The authors declare that the research was conducted in the absence of any commercial or financial relationships that could be construed as a potential conflict of interest.

## Publisher’s Note

All claims expressed in this article are solely those of the authors and do not necessarily represent those of their affiliated organizations, or those of the publisher, the editors and the reviewers. Any product that may be evaluated in this article, or claim that may be made by its manufacturer, is not guaranteed or endorsed by the publisher.

## Supplementary Material

Supplementary Figure 1 Detection of PHEV and Iba 1 in vary brain regions. Brain samples were harvested at 3-7 dpi and anatomically separated into 6 parts including olfactory bulb, cortex, hippocampus, thalamus, brainstem, and cerebellum. Total RNA was extracted to evaluate the viral load and Iba 1 expression.

Supplementary Figure 2 Optimization of PLX3397 treatment in mice. **(A)** Mice orally administrated with 200mg/kg or 290mg/kg as shown in . Morphological changes and counts of microglia were analyzed at -17 d, 7 d, and 0 d. Bars, 50 μm. **(B)** Body weight change. **(C)** Survival curves.

Supplementary Figure 3 Effection of Cd lipos i.v. injection on CNS microglial activation. Mice i.v. injected with Cd lipos as shown in . Morphological changes **(A)** and counts number **(B)** of microglia were detected at -2 d, 2 d, and 4 d. Bars, 50 μm. **(C)** Iba 1 mRNA expression was tested using qRT-PCR.
