# Supplementary figures and images for: Evidence of Microglial Immune Response Following Coronavirus PHEV Infection of CNS
Source: Front Immunol. 2022 Jan 10;12:804625. doi: 10.3389/fimmu.2021.804625 (PMC8784595; doi:10.3389/fimmu.2021.804625)

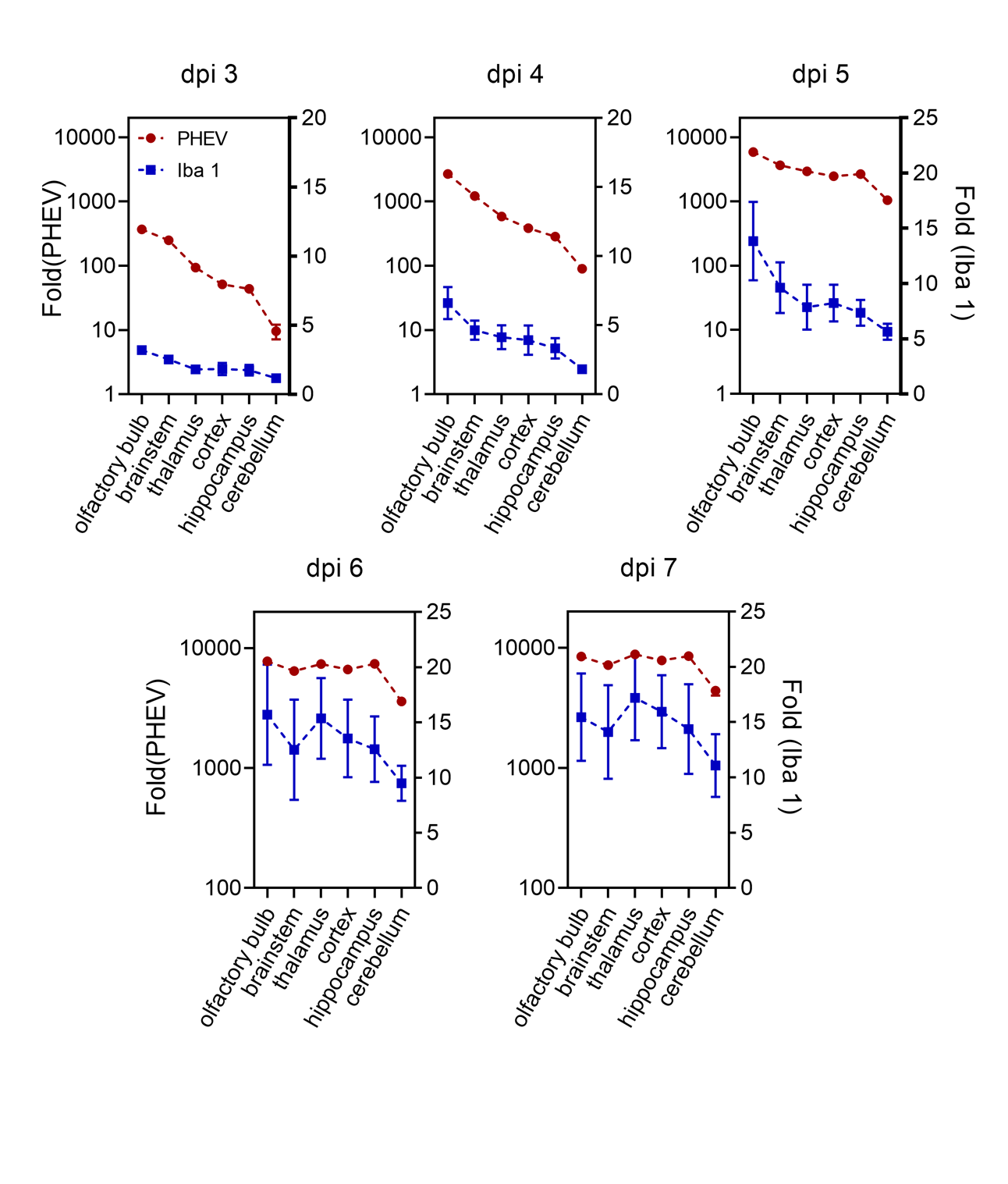

Supplement: Supplementary Figure 1 — Detection of PHEV and Iba 1 in vary brain regions. Brain samples were harvested at 3-7 dpi and anatomically separated into 6 parts including olfactory bulb, cortex, hippocampus, thalamus, brainstem, and cerebellum. Total RNA was extracted to evaluate the viral load and Iba 1 expression. [file Image_1.tif]

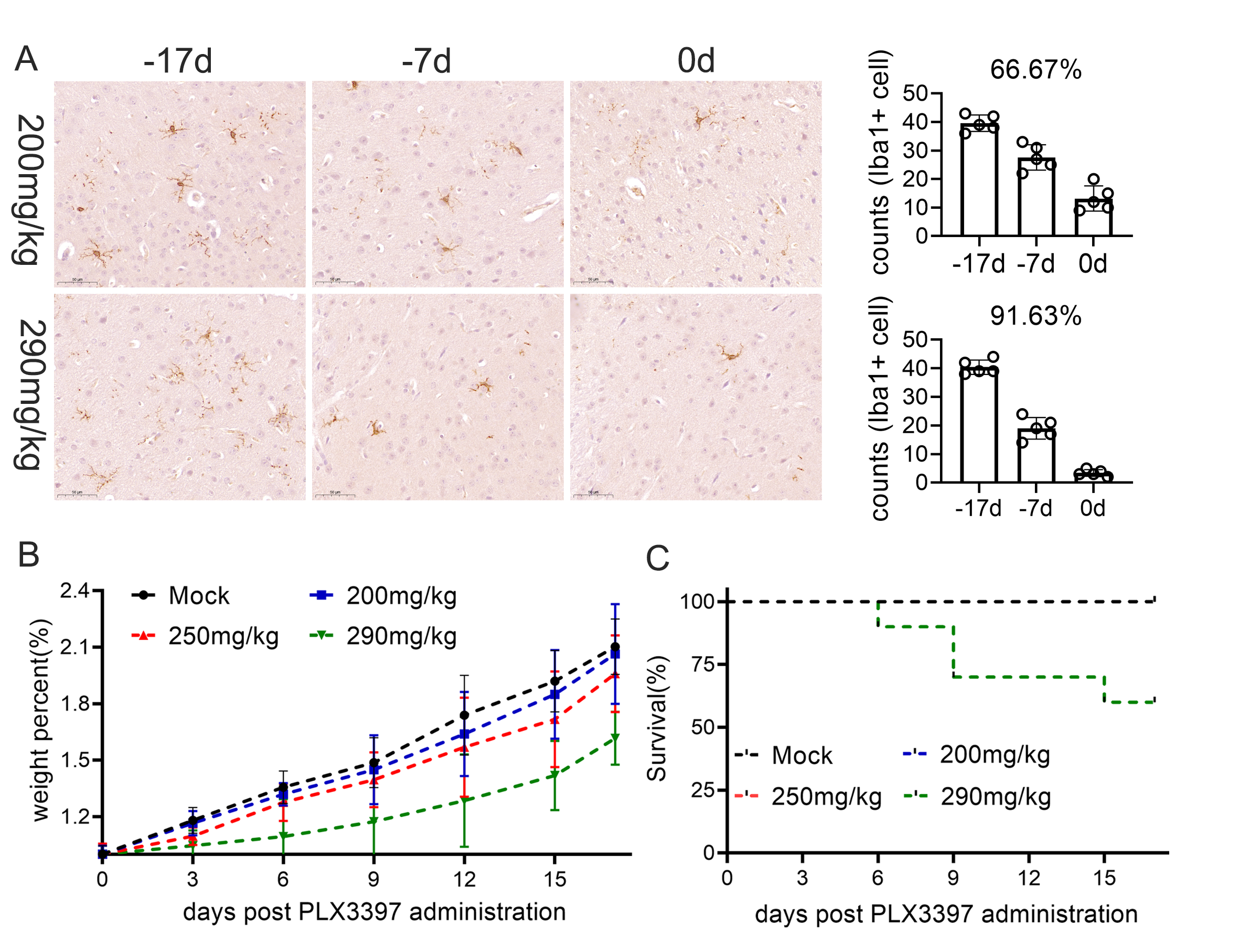

Supplement: Supplementary Figure 2 — Optimization of PLX3397 treatment in mice. (A) Mice orally administrated with 200mg/kg or 290mg/kg as shown in . Morphological changes and counts of microglia were analyzed at -17 d, 7 d, and 0 d. Bars, 50 μm. (B) Body weight change. (C) Survival curves. [file Image_2.tif]

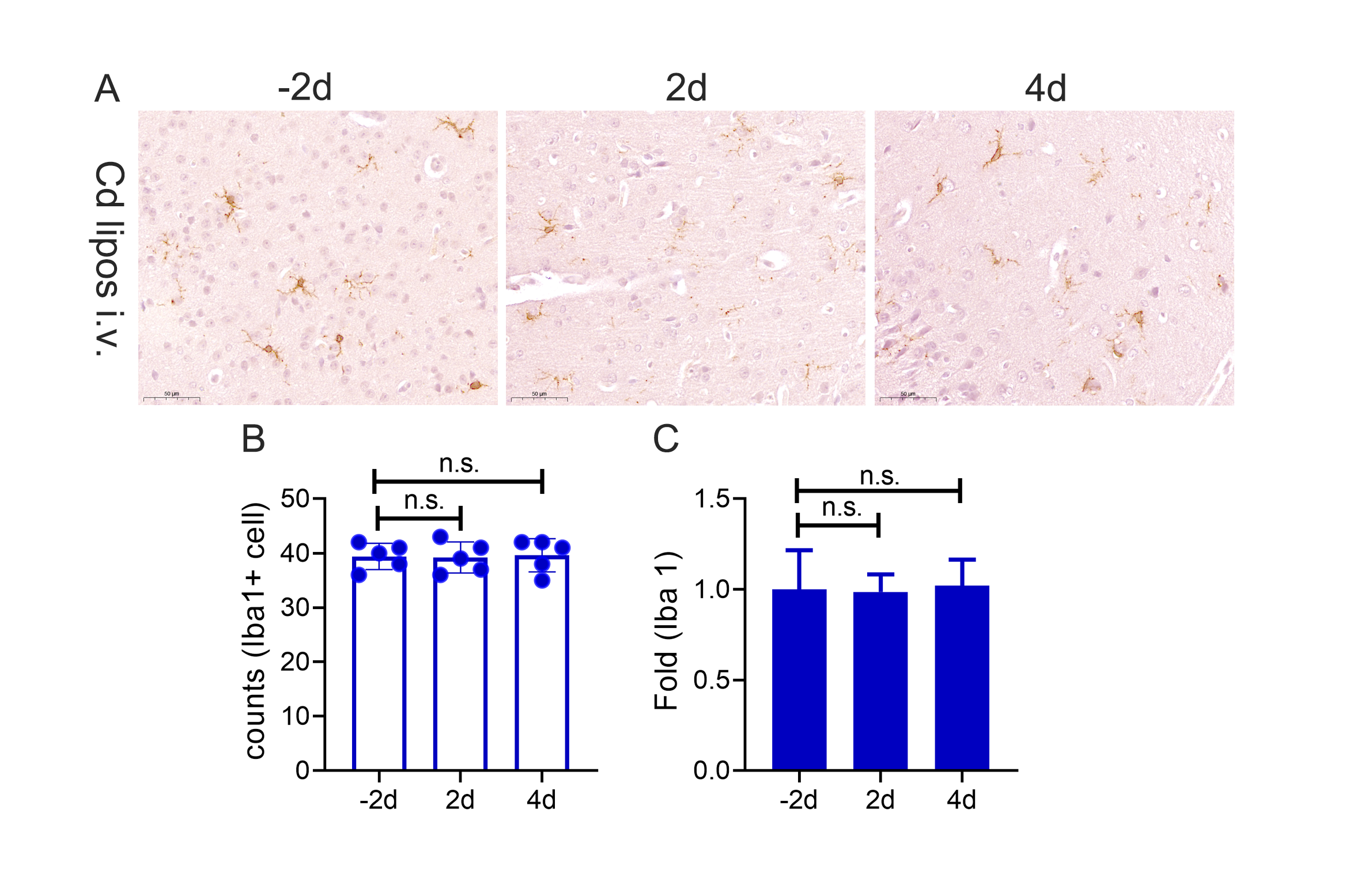

Supplement: Supplementary Figure 3 — Effection of Cd lipos i.v. injection on CNS microglial activation. Mice i.v. injected with Cd lipos as shown in . Morphological changes (A) and counts number (B) of microglia were detected at -2 d, 2 d, and 4 d. Bars, 50 μm. (C) Iba 1 mRNA expression was tested using qRT-PCR. [file Image_3.tif]
